# Microbial Biosynthesis of Medium-Chain-Length Polyhydroxyalkanoate (mcl-PHA) from Waste Cooking Oil

**DOI:** 10.3390/polym16152150

**Published:** 2024-07-29

**Authors:** Ahmed M. Elazzazy, Khawater Ali Abd, Noor M. Bataweel, Maged M. Mahmoud, Afra M. Baghdadi

**Affiliations:** 1 Department of Biological Sciences, College of Science, University of Jeddah, P.O. Box 80327, Jeddah 21589, Saudi Arabia; 1970136@uj.edu.sa (K.A.A.); amboghdadi@uj.edu.sa (A.M.B.); 2 King Fahad Medical Research Centre, King Abdulaziz University, Jeddah 21589, Saudi Arabia; no0ora.118@hotmail.com (N.M.B.); mamostafa@kau.edu.sa (M.M.M.); 3 Department of Medical Laboratory Sciences, Faculty of Applied Medical Sciences, King Abdulaziz University, Jeddah 21589, Saudi Arabia

**Keywords:** polyhydroxyalkanoates (PHAs), microbial biosynthesis, waste cooking oil, bioplastics, thermoplastic elastomers

## Abstract

Waste cooking oil is a common byproduct in the culinary industry, often posing disposal challenges. This study explores its conversion into the valuable bioplastic material, medium-chain-length polyhydroxyalkanoate (mcl-PHA), through microbial biosynthesis in controlled bioreactor conditions. Twenty-four bacterial isolates were obtained from oil-contaminated soil and waste materials in Mahd Ad-Dahab, Saudi Arabia. The best PHA-producing isolates were identified via 16S rDNA analysis as *Neobacillus niacini* and *Metabacillus niabensis*, with the sequences deposited in GenBank (accession numbers: PP346270 and PP346271). This study evaluated the effects of various carbon and nitrogen sources, as well as environmental factors, such as pH, temperature, and shaking speed, on the PHA production titer. *Neobacillus niacini* favored waste cooking oil and yeast extract, achieving a PHA production titer of 1.13 g/L, while *Metabacillus niabensis* preferred waste olive oil and urea, with a PHA production titer of 0.85 g/L. Both strains exhibited optimal growth at a neutral pH of 7, under optimal shaking -flask conditions. The bioreactor performance showed improved PHA production under controlled pH conditions, with a final titer of 9.75 g/L for *Neobacillus niacini* and 4.78 g/L for *Metabacillus niabensis*. Fourier transform infrared (FT-IR) spectroscopy and gas chromatography–mass spectrometry (GC-MS) confirmed the biosynthesized polymer as mcl-PHA. This research not only offers a sustainable method for transforming waste into valuable materials, but also provides insights into the optimal conditions for microbial PHA production, advancing environmental science and materials engineering.

## 1. Introduction

The urgent need for sustainable materials has increasingly led researchers to explore eco-friendly alternatives to conventional polymers, which are derived from petrochemical sources. Thermoplastic elastomer (TPE) is a polymer material that exhibits high elasticity in thermoset vulcanized rubber at room temperature and good processability at high temperatures [1]. Due to these unique properties, TPE is also known as a “third-generation rubber”. TPE is widely used across many industries including automotive, construction, household equipment, wires and cables, electronic products, food packaging, and medical equipment. Compared to traditional synthetic rubber, TPE has several benefits, including recyclability, light weight, flexibility, excellent compression set and flex fatigue, high- and low-temperature performance, good electrical insulation properties, heat and oil resistance, low permeability, and colorability [2]. Biopolymers with true rubber-like properties are rare among prokaryotic microorganisms. Microbial polyesters known as polyhydroxyalkanoates (PHAs) are biodegradable plastics [3]. Polyhydroxyalkanoates (PHAs) are biopolymers that have attracted considerable attention due to their biodegradability and physio-mechanical properties, which compare favorably with those of synthetic polymers. PHAs can be categorized into three types based on the length of their carbon side chains: short-chain, medium-chain, and long-chain PHAs. Short-chain PHAs, which include 4–5 carbon atoms, are known for their crystallinity and melting points, similar to polypropylene, specifically polyhydroxybutyrate (PHB). Medium-chain PHAs, such as poly(3-hydroxyhexanoate), exhibit lower tensile strength and melting points and are semicrystalline. In contrast, long-chain PHAs, with 14–25 carbon atoms like poly(3-hydroxyoctadecanoate), are rarely utilized in commercial applications due to their extended side chains [4]. Many species from Bacteria and Archaea domains produce PHA, such as *Cupriavidus necator, Bacillus* sp., *Pseudomonas* sp., *Halomonas* sp., and *Azotobacter* sp., that can accumulate PHAs as granules within their cells [5]. Moreover, mcl-PHA contains 3-hydroxy acid monomer units ranging in length from C6 to C14, i.e., alkanes, alkenes, free fatty acids, and triacylglycerols, and is produced by many bacterial species of *Pseudomonas* and *Bacillus* [6]. When provided with a substrate high in unsaturated fatty acids, such as soybean oil or linseed oil, mcl-PHA with a high concentration of side-chain olefinic groups is obtained. Depending on the olefin content, mcl-PHA has several potential applications, including biodegradable elastomers and adhesives [7]. Therefore, the search for novel biodegradable plastics is of increasing interest. The microbial production of TPEs offers a promising route toward sustainability, especially when utilizing waste materials as carbon and nitrogen sources [8]. Further fine-tuning of the production process can be achieved through manipulation of the bioreactor’s environmental conditions, such as varying the temperature and pH level, to optimize microbial metabolic pathways for TPE synthesis. By employing synthetic biology techniques, researchers have been successful in engineering bacteria to produce TPEs with mechanical properties that compete with those of their petrochemical counterparts [9]. In this study, we aimed to explore the bacterial production of the thermoplastic elastomer polyhydroxyalkanoate (mcl-PHA) using multiple types of waste cooking oils (WCOs) as both carbon and nitrogen sources, as well as the effects of different bioreactor conditions, such as temperature and pH.

## 2. Materials and Methods

### 2.1. Microbial Strains and Culture Conditions

Soil samples were procured from distinct regions of Mahd Ad-Dahab in the Kingdom of Saudi Arabia. Four samples were gathered from areas polluted with automotive oil residues, two samples were obtained from zones contaminated with miscellaneous waste materials, and six samples were collected from various mining sites. All samples were acquired from a soil depth ranging between 2 and 5 cm, using aseptic techniques, and were immediately transferred to sterile containers for transportation. Upon arrival at the laboratory, 1 g of each soil sample was inoculated into an enrichment culture, using a mineral salt medium (MSM). The MSM contained the following components per liter: (NH_4_)_2_SO_4_ 3.0 g, Na_2_SO_4_ 3.2 g, KCl 0.1 g, K_2_HPO_4_ 0.05 g, Ca(NO_3_)_2_ 0.5 g, MgSO_4_ 0.01 g, and 10 g glucose per liter. These cultures were then incubated in a shaking incubator at 37 °C. The samples from the Mahd Al-Dahab mining sites were incubated for a prolonged period of 6 days to facilitate bacterial isolation. Subsequently, 0.1 mL aliquots from each enriched culture were subjected to serial dilution in 0.85% normal saline. These dilutions were subsequently plated onto nutrient agar media, using the spread plate technique. After the bacterial colonies were successfully isolated and purified, the bacteria were preserved as frozen stocks in nutrient broth supplemented with 50% glycerol and stored at −20 °C for future experimental use.

### 2.2. Screening of mcl-PHA-Producing Bacterial Isolates

To evaluate intracellular mcl-PHA accumulation in bacterial isolates, we utilized Nile red and Sudan Black B staining, followed by confocal fluorescence microscopy, as adapted from [10]. After 72 h of incubation, 10 mL of cell culture at the late exponential growth phase (OD_600 nm_ = 1.50) was transferred into a new flask for Nile red staining. Nile red (Sigma-Aldrich, St. Louis, MO, USA) was dissolved in methanol to create a 500 µg/mL stock solution. A stock dilution of 1:1000 was prepared in the growth medium (final concentration of 0.5 µg/mL) and incubated for 24 h at 42 °C with shaking. The stained cells (OD600 nm = 1.95) were harvested by centrifugation, washed with 0.85% saline buffer, and resuspended for confocal microscopy analysis. For the microscopy, a drop of the stained cell suspension was placed on a slide, covered with a cover slip, and sealed with nail polish. The cells were imaged using a Leica TCS SP8 confocal microscope with a HC PL APO CS2 63×/1.3 glycerol objective. The cells were excited by a 553 nm Ar-laser for Nile red and detected with a Leica HyD hybrid detector (569 nm–648 nm). Concurrently, Sudan Black B was employed as a qualitative marker for PHA synthesis. Bacterial isolates were cultured on nutrient agar plates, enriched with 1% weight/volume (*w*/*v*) glucose, and incubated at a constant temperature of 37 °C for a duration ranging from 24 to 48 h. After incubation, the bacterial colonies were treated with a 0.03% (*w*/*v*) Sudan Black B solution, which was evenly spread across the plate. The plates were then allowed to stand undisturbed at room temperature for 30 min. Subsequently, 96% ethanol was used to rinse off any excess staining solution. The presence of PHA was inferred based on the retention of Sudan Black B dye by the bacterial colonies.

### 2.3. Characterization and Identification of Test Isolates

The method used for characterizing and identifying the test isolates involved two main steps: DNA isolation and 16S rRNA gene amplification using PCR. The genomic DNA was extracted according to the standard protocol described by [11], the bacterial strains were collected and subjected to a series of procedures. This included mixing the strains with TES buffer, lysozyme, and proteinase K to break down the cell structures and release the genomic DNA. After incubation and the addition of sodium acetate and chloroform isoamyl, the mixture was centrifuged to separate the upper zone containing DNA. The DNA was then precipitated with isopropanol, stored overnight, and further processed the next day. Following purification and resuspension, the isolated DNA was loaded onto an agarose gel for electrophoresis and visualization [12]. In the second step, full-length 16S rDNA sequences were amplified by PCR using primers 27F (50-AGA GTT TGA TCM TGG CTC AG-30) and 1492R (50-TAC GGY TAC CTT GTT ACG ACT T-30). The PCR products were then electrophoresed on an agarose gel and the bands were visualized. Subsequent purification and sequencing of the PCR amplicons were carried out. The obtained DNA sequences were compared with the GenBank database using BLASTn to identify the closest matches. Finally, phylogenetic analyses were performed using MEGA 6.1 software to determine the evolutionary relationships of the bacterial strains based on their genetic sequences. Overall, this method allowed for the characterization and identification of the test isolates based on their DNA profiles and phylogenetic analysis [13].

### 2.4. Extraction, Characterization, and Quantification of mcl-PHA

For the quantitative screening of PHA, 5% of pre-culture broth was inoculated into 100 mL of MSM medium and incubated for 48 h at 35 °C. Post-incubation, 10 mL of the culture was centrifuged at 8000 rpm for 15 min to collect the cell pellet. This pellet was treated with sodium hypochlorite (NaOCl) at 50 °C for 1 h. Following this treatment, the pellet was centrifuged at 12,000 rpm for 30 min. The pellet underwent a series of washes: first with distilled water to remove the bleach solution, followed by acetone to eliminate low-molecular-weight lipids, and finally with absolute ethanol. The cleaned pellet was then dissolved in 10 mL of chloroform (CHCl_3_) and incubated overnight at 50 °C. The resulting solution was allowed to dry at room temperature, stored at 4 °C, and weighed [14].

Characterization of mcl-PHA:

- Fourier Transform Infrared Spectroscopy (FTIR): The functional groups present in the extracted PHA were evaluated using an FT-IR spectrometer (PerkinElmer, Cambridge, UK). Briefly, 1 mg of PHA was diluted in 7 mL of chloroform and a drop of the mixture was overlaid on an FT-IR KBr disk. The infrared (IR) spectra were recorded in the range of 400–4000 cm^−1^, at a spectral resolution of 4 cm^−1^, under vacuum pressure.
- Gas Chromatography–Mass Spectrometry (GC-MS): The monomer composition of the mcl-PHA was determined using GC-MS (Agilent Technologies, Cheadle, UK), equipped with a gas chromatograph (7890B) and a mass spectrometer detector (5977A). Approximately 10 mg of the extracted PHA was methanolized with 2 mL of methanol, containing 15% (*v*/*v*) sulfuric acid and 2 mL of chloroform, at 100 °C for 140 min. After cooling, 1 mL of deionized water was added, and the mixture was vortexed and allowed to separate. The organic phase was analyzed by GC-MS, equipped with a capillary column (30 m × 0.25 mm × 0.25 µm).
- Quantification of mcl-PHA: The mcl-PHA content in the bacterial biomass was quantified by gravimetric analysis [15]. The dried PHA granules obtained after precipitation and drying were weighed, and the PHA content was expressed as a percentage of the cell dry weight (CDW). The percentage titer of the PHA was calculated using the following formula. The intracellular PHA accumulation was assessed as the percentage of the PHA in relation to the dry cell weight and was calculated as follows:

$$\mathrm{PHA}\;\mathrm{accumulation}\;(\%)=\frac{\mathrm{Dry}\;\mathrm{weight}\;\mathrm{of}\;\mathrm{extracted}\;\mathrm{PHA}\;(g/L)\;\times \;100}{\mathrm{DCW}\;(g/L).}$$

### 2.5. Optimization of Cultural Parameters for Maximum mcl-PHA Production

#### 2.5.1. Effects of Different Carbon Sources on Growth and mcl-PHA Production

The impact of various carbon sources on both the growth and polyhydroxyalkanoate (PHA) production of the bacterial culture was investigated. A 100 mL volume of sterilized nutrient broth media (NB) was enriched with different carbon sources, waste cooking oils (olive, palm, and sunflower oils), olive oil, potato starch, glucose, and glycerol at a concentration of 20 g/L. The composition of the nutrient broth was as follows (g/L): peptone, 5; NaCl, 5; yeast extract, 1.5; beef extract 1.5. To prepare 100 mL of nutrient broth, 1.3 g of nutrient broth powder (purchased from Hi-Media, Thane, India) was suspended in 100 mL of distilled water. The pH of each medium was adjusted to 7.0 using sodium phosphate buffer saline (PBS). The pure bacterial isolate was inoculated in each well, after which the mixture was subsequently incubated at 37 °C in an orbital incubator (New Brunswick Innova 44, Eppendorf, Hamburg, Germany) at 150 rpm for 24 h. Measurements of both the biomass and PHA content were taken from the culture broth after 48 h of incubation.

#### 2.5.2. Effects of Different Nitrogen Sources on Growth and mcl-PHA Production

The influence of various nitrogen sources on the growth and polyhydroxyalkanoate (PHA) production of bacterial cultures was examined. Experiments were conducted using 100 mL of sterilized nutrient broth media (NB) supplemented with an optimal carbon source at a concentration of 20 g/L, as determined by prior studies [16,17]. Specifically, ammonium chloride, ammonium molybdate, yeast extract, and urea were added at a concentration of 2 g/L. Additionally, 2 g/L of peptone served as a stable nitrogen source. The pH of each medium was standardized to 7.0. The cultures were incubated at 37 °C and agitated at a speed of 150 rpm. After 48 h of incubation, both the biomass and mcl-PHA content were quantified in the cell culture broth.

#### 2.5.3. Effect of Varied pH on Growth and mcl-PHA Production

Bacterial isolates were cultured in 100 mL of nutrient broth media (NB) optimized with a carbon source at a concentration of 20 g/L and a nitrogen source at 2 g/L, as determined by previous studies. The media were adjusted to varying pH levels (3, 5, 7, 9, and 11). The inoculated flasks were then incubated at 37 °C, with agitation at 150 rpm for 48 h, after which the polyhydroxyalkanoate (PHA) production was quantified. Using the same NB composition and pH level optimized by prior experiments, bacterial isolates were cultured in 100 mL of media. The cultures were incubated at different temperatures, specifically 20 °C, 37 °C, and 45 °C, while being agitated at 150 rpm. After 48 h of incubation, the PHA content in the cultures was assessed.

### 2.6. Bioreactor Cultivation Procedure

The cultivation was conducted in a 10 L tabletop bioreactor (New Brunswick Scientific, Edison, NJ, USA), where the parameters, such as dissolved oxygen, temperature, and pH, were regulated. Oxygen was manually supplied at a rate of 100% (or 1.0 *v*/*v*)) using a flow meter. Samples were periodically collected via a sampling system attached to the bottom of the bioreactor for the determination of the cell dry weight and PHA content. The temperature was maintained at 37 °C, while the dissolved oxygen concentration was regulated between 0 and 100%. Upon the completion of batch cultivation, measurements were taken for the dissolved oxygen percentage, pH, residual carbohydrate content (g/L), PHA content, and cell dry weight (CDW; g/L).

### 2.7. Analytical Techniques

#### 2.7.1. Estimation of Cell Dry Weight

For each sample, 50 mL of the sample was transferred to a conical tube and centrifuged at speeds between 6000 and 8000× *g*. The supernatant was discarded and the remaining pellet was air-dried at 105 °C until a constant weight was achieved (Numak DHG-9053A). The dry weight was determined by comparing the weight of the conical tube before and after centrifugation.

#### 2.7.2. pH Regulation

A glass electrode (Ingold) was used for the pH measurements. The pH was uncontrolled during the first batch of cultivation, whereas in the second batch, it was regulated throughout the process.

### 2.8. Production Kinetics

The fermentation kinetic parameters were studied by calculating the biomass yield related to the substrate consumption *Y*_X/S_ (g/g), the product yield with respect to the substrate consumption *Y*_P/S_ (g/g), and the volumetric productivity *P*_PHA_ (g/L/h) and product yield with respect to the fermentation time (h). The following equation was used to calculate the percentage yield of bacteriocin:

1. PHA volumetric productivity (*P_v_*):

$$Productivity\;({g/L}^{/}h)=\frac{PHA(g/L)}{Time\;(h)}$$

2. Biomass volumetric productivity (*B_v_*):

$$Biomass\;({g/L}^{/}h)=\frac{CDW(g/L)}{Time\;(h)}$$

3. Yield of biomass from the substrate (*Y_x/s_*):

$$Y\;(X/S)=\frac{CDW(g)}{Substrate\;consumed\;(g)}$$

4. Yield of PHA from the substrate (*Y_P/s_*):

$$Y\;(P/S)=\frac{PHA(g)}{Substrate\;consumed\;(g)}$$

5. Specific growth rate (µ):

$$\mu =\frac{ln\left(N_{t}\right)-ln(N_{0})}{t-t_{0}}$$

where *N_t_* and *N*_0_ are the cell density at time *t* and *t*_0_, respectively.

### 2.9. Data Collection and Analysis

All experiments included triplicate samples for each independent variable, such as the bacterial isolate, carbon source, and nitrogen source, to ensure the reliability of the results. Overall, the statistical design aimed to rigorously evaluate the variables under consideration and their impact on bacterial growth and mcl-PHA production.

## 3. Results

### 3.1. Strain Isolation, Screening, and Dentification

**Figure 1:**
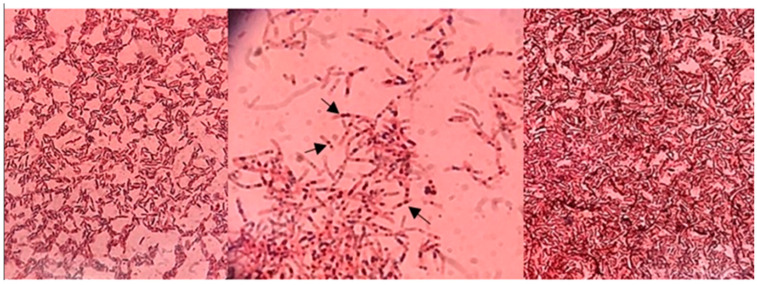
PHA-producing bacterial strains under a light microscope after staining with Sudan Black B dye; arrows indicate the PHA granules accumulated inside the bacteria.

**Figure 2:**
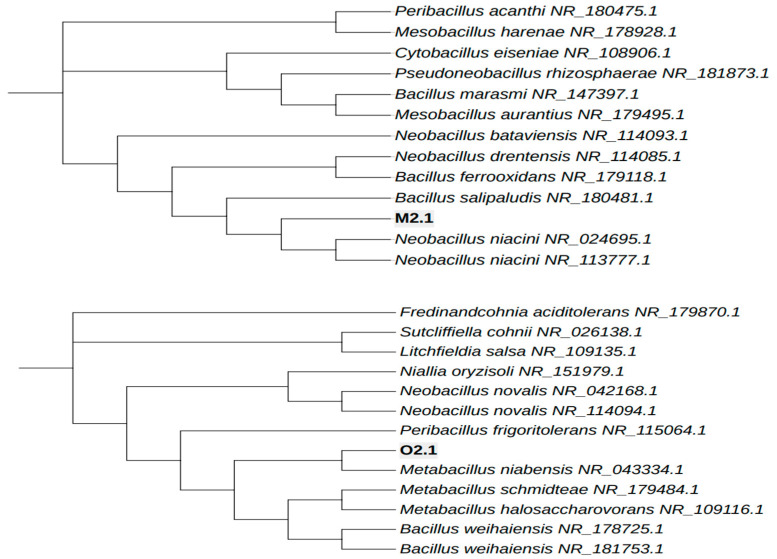
Phylogenetic tree showing the positions of the PHA-producing bacterial strains *Neobacillus niacini* (M2.1) and *Metabacillus niabensis* (O2.1), along with their closest related strains based on 16S rRNA gene sequences.

Screening for polyhydroxyalkanoate (PHA) production was performed using the Nile red and Sudan Black B staining methods. Of the 24 bacterial isolates tested, two demonstrated PHA-producing potential, as indicated by a colorimetric change to dark blue after staining, which is visible under a light microscope (Figure 1, Figures S1 and S2). The arrows in Figure 1 highlight the PHA granules accumulated inside the bacteria. Phylogenetic analysis was conducted using 16S rDNA sequence data, employing NCBI MegaBLAST for molecular identification. A neighbor-joining tree (Figure 2) was constructed for visualization. The two isolates, M2.1 and O2.1, which exhibited the highest PHA production capacity, were taxonomically identified through 16S rDNA sequencing as *Neobacillus niacini* (accession number: PP346270) and *Metabacillus niabensis* (accession number: PP346271), respectively. 

### 3.2. Cultivation Conditions Optimization

**Figure 3:**
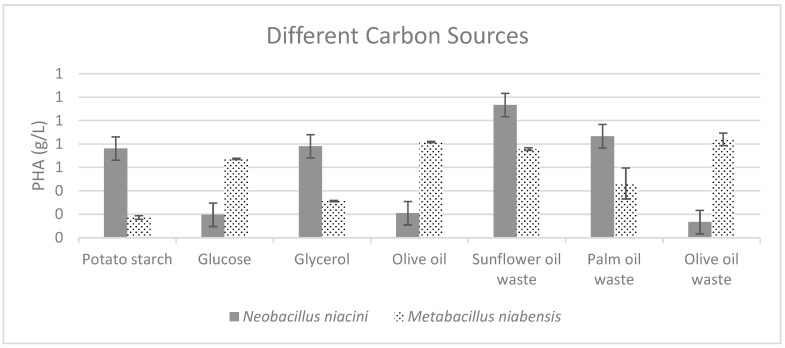
Effect of different carbon sources on the production of PHA by PHA-producing bacterial strains; where the vertical axis represents the mass of produced PHA (g) from 1 g of dried cells.

The efficacy of various carbon sources on cell growth and PHA production in two bacterial strains, *Neobacillus niacini* and *Metabacillus niabensis*, was studied. In *Neobacillus niacini*, sunflower oil waste was the most effective in increasing the total PHA titer, achieving 1.1 g/L, suggesting that it could be ideal for maximizing both PHA production and cell growth. Conversely, *Metabacillus niabensis* preferred waste olive oil, which resulted in the highest total PHA titer of 0.82 g/L (Figure 3).

**Figure 4:**
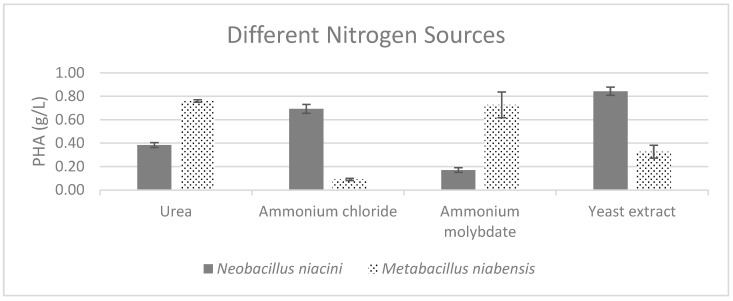
Effect of different nitrogen sources on the production of PHA by PHA-producing bacterial strains; where the vertical axis represents the weight of produced PHA (g) from 1 g of dried cells.

The nitrogen sources were also examined, with yeast extract being the most suitable nitrogen source for *Neobacillus niacini* and urea leading to the highest PHA weight for *Metabacillus niabensis* (Figure 4).

**Figure 5:**
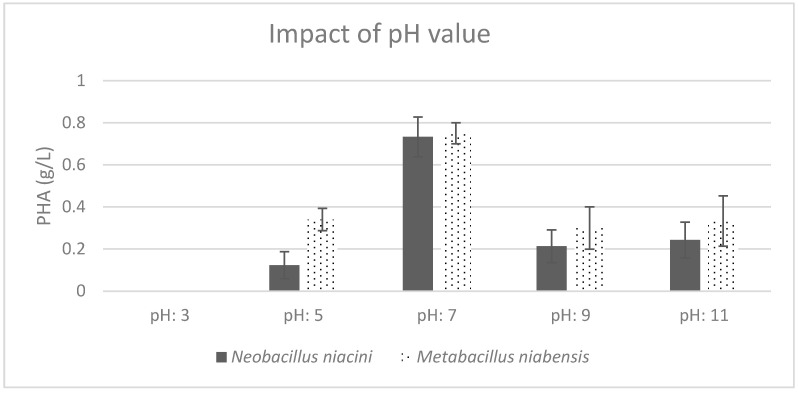
Effect of different pH values on the production of PHA by PHA-producing bacterial strains; where the vertical axis represents the weight of produced PHA (g) from 1 g of dried cells.

Both strains showed a preference for a neutral pH of 7, which is universally favorable for promoting cell growth and PHA production. Acidic conditions, with a pH of 3, were found to inhibit the growth of both strains (Figure 5).

**Figure 6:**
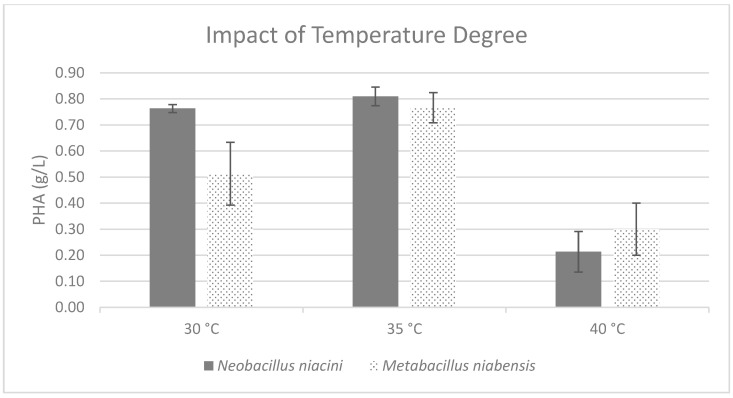
Effect of different temperatures on the production of PHA by PHA-producing bacterial strains; where the vertical axis represents the weight of produced PHA (g) from 1 g of dried cells.

**Figure 7:**
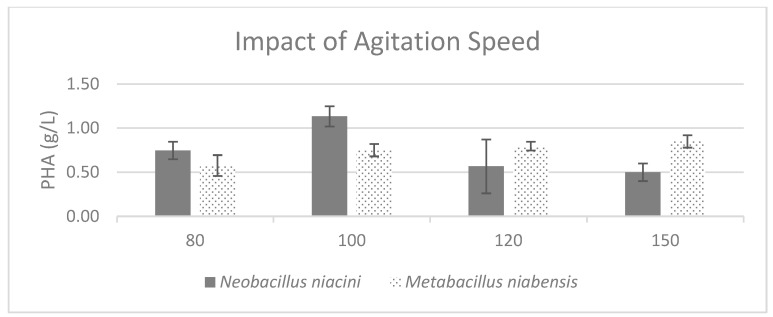
Effect of different agitation speeds on the production of PHA by PHA-producing bacterial strains; where the vertical axis represents the weight of produced PHA (g) from 1 g of dried cells.

Temperature was found to be crucial for both strains, with both *Neobacillus niacini* and *Metabacillus niabensis* achieving optimal results at 35 °C (Figure 6). The shaking speed variable revealed interesting results, with *Neobacillus niacini* exhibiting optimal performance at 100 rpm, while *Metabacillus niabensis* demonstrated flexibility regarding the optimal shaking speed, with 150 rpm being ideal for maximizing both the cell and PHA titer (Figure 7).

Table 1 summarizes the PHA titer under various optimum experimental conditions for *Neobacillus niacini* and *Metabacillus niabensis*. The highest PHA titer for *Neobacillus niacini* was achieved using sunflower oil as the carbon source, yeast extract as the nitrogen source, at pH 7, 35 °C, and an agitation speed of 100 rpm. In contrast, *Metabacillus niabensis* exhibited the highest PHA titer with waste olive oil as the carbon source, urea as the nitrogen source, at pH 7, 35 °C, and an agitation speed of 150 rpm.

#### Batch Cultivation and Production 

**Figure 8:**
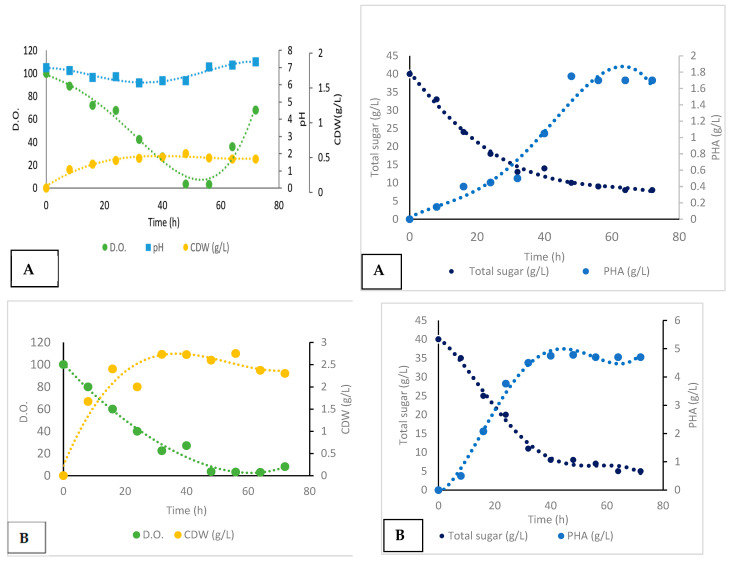
(**A**,**B**) Bioreactor performance analysis for *Metabacillus niabensis*: (**A**) uncontrolled pH conditions and (**B**) controlled pH conditions. The analysis tracks key parameters, such as pH, dissolved oxygen (D.O.), PHA productivity, substrate consumption, and dry weight, over different time phases.

**Figure 9:**
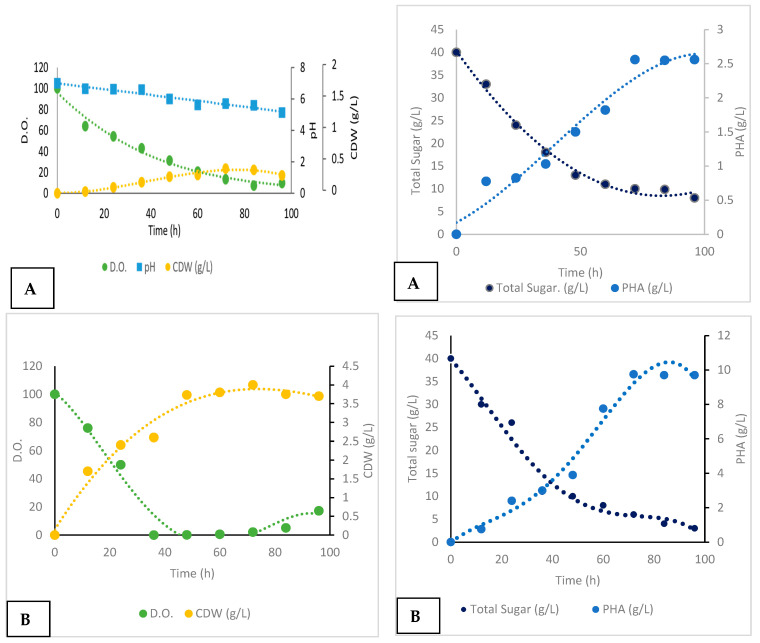
(**A**,**B**) Bioreactor performance analysis for *Neobacillus niacini*: (**A**) uncontrolled pH conditions and (**B**) controlled pH conditions. The analysis tracks key parameters, such as pH, dissolved oxygen (D.O.), PHA productivity, substrate consumption, and dry weight, over different time phases.

The bioreactor performance for the two bacterial strains, *Metabacillus niabensis* and *Neobacillus niacini*, was analyzed by tracking key parameters, such as the dissolved oxygen (D.O.), total carbon or sugar concentration, and dry weight, over different time points, under controlled and uncontrolled pH conditions. For *Metabacillus niabensis* with an uncontrolled pH (Figure 8A): The pH fluctuated significantly, ranging from 7.5 to 5.0; the dissolved oxygen (D.O.) levels decreased sharply from 100% to 3.2% by 72 h; the substrate consumption decreased from 40 g/L to 5 g/L by 72 h; and the PHA concentration peaked at 0.75 g/L around 40 h, but decreased to 0.7 g/L by 72 h. While with a controlled pH (Figure 8B): A stable pH was maintained around 7.0 throughout the experiment; The D.O. levels remained more stable, with a final value of around 8.1%; the substrate consumption was steady, reducing from 40 g/L to 8 g/L by 72 h; and the PHA concentration consistently increased to 0.75 g/L by 40 h and remained stable. The data suggest that *Metabacillus niabensis* has a higher growth rate and metabolic activity compared to *Neobacillus niacini*, but both strains are effective in producing PHA, with differing production patterns and growth dynamics (Figure 8A,B and Figure 9A,B).

The experimental results for *Neobacillus niacini* with an uncontrolled pH (Figure 9A): The pH varied significantly from 7.0 to 5.0; the D.O. levels dropped from 100% to 3.2% by 72 h; the substrate consumption reduced from 40 g/L to 5 g/L by 72 h; and the PHA concentration increased to 0.75 g/L by 40 h and decreased to 0.7 g/L by 72 h. However, with the controlled pH (Figure 9B): The pH was maintained around 7.0 throughout the experiment; The D.O. levels remained relatively stable, with a final value of around 17.2%; the substrate consumption decreased steadily from 40 g/L to 3 g/L by 72 h; and the PHA concentration consistently increased to 1.0 g/L by 48 h and remained stable.

Table 2 compares the fermentation kinetics of *Neobacillus niacini* under controlled and uncontrolled pH conditions. Under uncontrolled pH conditions, the PHA volumetric productivity was 0.035 g/L/h, the biomass volumetric productivity was 0.02 g/L/h, the biomass yield from the substrate (Y_x/S_) was 0.15 g/g, and the PHA yield from the substrate (Y_p/S_) was 0.25 g/g. The final PHA concentration was 1.75 g/L, with a CDW of 2.0 g/L, and a specific growth rate of 0.07 h^−1^. Under controlled pH conditions, the PHA volumetric productivity increased to 0.13 g/L/h, the biomass volumetric productivity was 0.05 g/L/h, the biomass yield from the substrate (Y_x/S_) was 1.62 g/g, and the PHA yield from the substrate (Y_p/S_) was 0.6 g/g. The final PHA concentration was significantly higher at 9.75 g/L, with a CDW of 4.0 g/L, and a specific growth rate of 0.08 h^−1^.

Table 3 presents the fermentation kinetics of *Metabacillus niabensis* under controlled and uncontrolled pH conditions. For the uncontrolled pH batch, the PHA volumetric productivity was 0.036 g/L/h, the biomass volumetric productivity was 0.04 g/L/h, the biomass yield from the substrate (Y_x/S_) was 0.2 g, and the PHA yield from the substrate (Y_p/S_) was 0.17 g/g. The final PHA concentration was 1.75 g/L, with a CDW of 2.0 g/L, and a specific growth rate of 0.11 h^−1^. For the controlled pH batch, the PHA volumetric productivity was 1.0 g/L/h, the biomass volumetric productivity was 0.054 g/L/h, the biomass yield from the substrate (Y_x/s_) was 0.32 g/g, and the PHA yield from the substrate (Y_p/S_) was 0.6 g/g. The final PHA concentration was 4.78 g/L, with a CDW of 2.6 g/L, and a specific growth rate of 0.11 h^−1^.

### 3.3. Characterization of Produced PHAs

**Figure 10:**
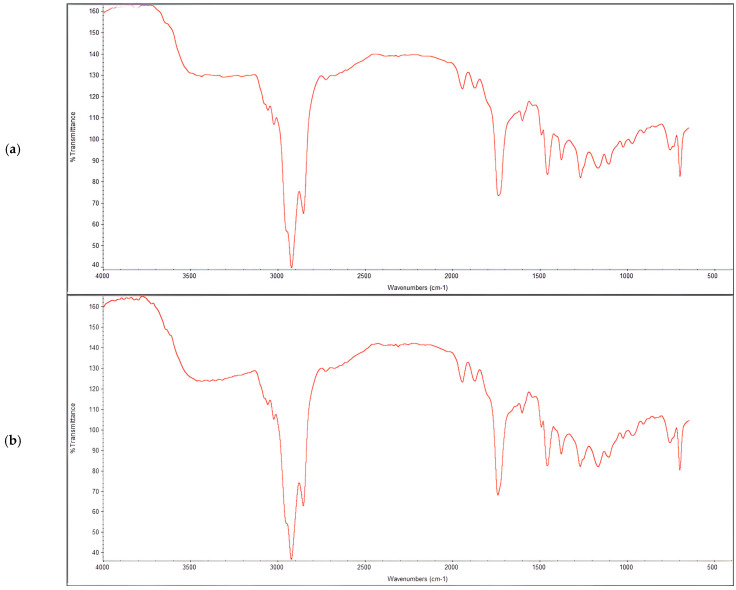
(**a**,**b**). Obtained FT-IR spectrum of plastic samples extracted from isolates *Neobacillus niacin* and *Metabacillus niabensis*.

FT-IR spectroscopy was used to characterize the functional groups in the PHA polymers derived from *Metabacillus niabensis* and *Neobacillus niacini*. The spectrum showed distinct peaks corresponding to various functional groups. The presence of the hydroxyl group was confirmed by a peak at 3436 cm^–1^, associated with O–H stretching vibrations. Peaks at approximately 2970 cm^–1^ indicated asymmetric CH3 stretching, which may interact with the carbonyl (C=O) group, forming a C–H–O linkage. The peak at 2921 cm^–1^ was attributed to asymmetric methylene (CH_2_) groups, which contributed to the lateral chain assembly of the monomeric units. The presence of amide groups was detected, with amide I (-CO-N-) observed near 1561 cm^–1^ and amide II (N–H) near 1414 cm^–1^. The terminal CH3 group was identified by a peak near 1018 cm^–1^. Additional bands at 2983, 1561, 1414, 1018, and 599 cm^−1^ were attributed to the stretching and bending vibrations of -CH2 and -CH, -OH, and C–O bonds in the copolymer. These findings led us to conclude that the extracted polymer was indeed mcl-PHA (Figure 10a,b).

**Figure 11:**
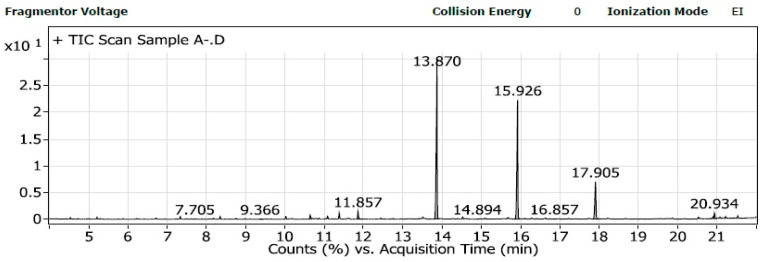
Gas chromatography–mass spectrometry (GC-MS) of extracted PHA from *Neobacillus niacini.*

**Figure 12:**
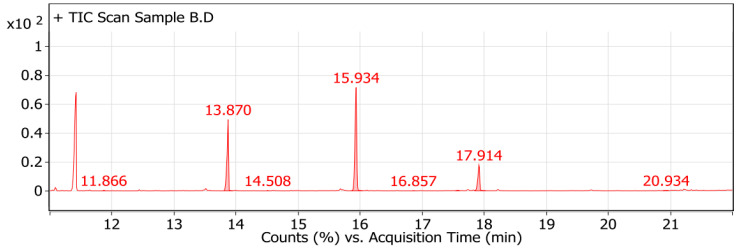
Gas chromatography–mass spectrometry (GC-MS) of extracted PHA from *Metabacillus niabensis*.

The GC-MS analysis of Sample A (Figure 11), produced from *Neobacillus niacini*, revealed various medium-chain fatty acid esters, including nonanoic acid, methyl ester (~172 g/mol); heptanedioic acid, dimethyl ester (~174 g/mol); and octanedioic acid, dimethyl ester (~188 g/mol). Other components included tridecanoic acid, 12-methyl-, methyl ester (~256 g/mol); methyl 9-methyltetradecanoate (~270 g/mol); hexadecanoic acid, methyl ester (~270 g/mol); heptadecanoic acid, methyl ester (~284 g/mol); oxiraneoctanoic acid, 3-octyl-, methyl ester (~314 g/mol); and octadecanoic acid, 9,10-dihydroxy-, methyl ester (~330 g/mol). For Sample B (Figure 12), produced from *Metabacillus niabensis*, the analysis identified nonanedioic acid, dimethyl ester (~200 g/mol); tridecanoic acid, 12-methyl-, methyl ester (~256 g/mol); methyl 9-methyltetradecanoate (~270 g/mol); hexadecanoic acid, methyl ester (~270 g/mol); heptadecanoic acid, 16-methyl-, methyl ester (~284 g/mol); 13-octadecenoic acid, methyl ester (~296 g/mol); and oxiraneoctanoic acid, 3-octyl-, methyl ester (~314 g/mol). The identified components suggest that Sample A includes a broad mixture of fatty acid methyl esters, typical of bioplastics, while Sample B aligns more closely with medium-chain-length polyhydroxyalkanoates (mcl-PHAs), due to the presence of specific medium-chain acids and esters. Additionally, Figure S3 shows the extraction process of PHA from bacterial strains: (a) PHA sheets produced by *Neobacillus niacini*, (b) PHA sheets produced by *Metabacillus niabensis*, and (c) PHA powder after collection.

## 4. Discussion

Extremophiles have garnered considerable interest for their ability to produce metabolites, such as biodegradable polymers, which are valuable for industrial applications [18]. Therefore, this research aimed to assess the ability to produce polyhydroxyalkanoates from diverse soil samples contaminated with oil residues, waste materials, and mining residues from extreme habitats in Mahd Ad-Dahab, Saudi Arabia. This geographical diversity is crucial because it could lead to the identification of novel bacterial strains optimized for waste conversion, thus enriching the existing microbial libraries [18]. The use of Nile red and Sudan Black B staining methods for PHA screening is a well-established technique for detecting bioplastics [19]. Through the extensive screening of more than 12 samples from these harsh regions, among these samples, only two isolates were identified as potential producers of polyhydroxyalkanoates (PHAs). These isolates were examined and characterized based on their molecular properties. Notably, both bacterial isolates were categorized as the Bacillus genus. The most promising isolates were identified as *Neobacillus niacini* and *Metabacillus niabensis*, using a 16S rDNA sequencing approach. This finding aligns with previous research that identified Bacillus as a prominent player in PHA synthesis [20]. Specifically, bacteria from the Bacillus genus are known to produce the class IV PHA synthase enzyme, which plays a vital role in the creation of polyhydroxyalkanoates, as highlighted in previous research [21].

In this study, a methodical approach, focusing on one factor at a time in shaking flask conditions, was employed to investigate how different variables in the modified minimal medium influenced PHA production by the isolated strains. It was found that fine-tuning these cultivation parameters significantly affected the rate at which PHA was produced [22]. This study also revealed that sunflower oil waste was the most effective carbon source for increasing the total PHA titer in *N. niacini*. Conversely, olive oil waste was the best carbon source for maximizing the total PHA titer in *M. niabensis*. Yeast extract was found to be the most suitable nitrogen source for *N. niacini*, while urea was the most effective nitrogen source for *M. niabensis*. These findings are consistent with previous studies that investigated the effect of different carbon and nitrogen sources on PHA production by various bacterial strains [23,24]. Jiang et al. [25] determined that PHA production substrates from different carbon sources fall into three categories: carbohydrates (simple sugars), triacylglycerols (oils and fats from plants and animals), and hydrocarbons (waste plastics). In bacterial cells, PHA biosynthesis substrates are usually small molecules due to the thick, rigid cell walls that prevent large molecules from entering, requiring external transformation for their use. The type and composition of the PHAs produced depend on the substrate specificity of the PHA synthase, carbon sources, and active metabolic pathways in the cells [26,27]. Nitrogen is essential for microbial growth, with different PHA-producing strains preferring various nitrogen sources, such as ammonia, urea, yeast extract, and nitrate [28]. Yeast extract was found to be the most effective nitrogen source for PHA production in *Neobacillus niacini* and *Metabacillus niabensis*, potentially due to its complex nutritional profile, which enhances PHA biosynthesis pathways. A complex nutritional profile provides a balanced supply of essential nutrients and micronutrients, which supports robust cell growth and metabolic activity. This, in turn, creates favorable conditions for the accumulation of PHA as a storage polymer [27]. This study suggests that optimizing PHA production from waste materials could be achieved using *Neobacillus niacini* and *Metabacillus niabensis*. Yeast extract was identified as the most effective nitrogen source for PHA production in these bacterial strains, leading to a PHA content of 55.2%. This enhanced yield is likely due to the rich nutritional profile of yeast extract, which promotes various metabolic pathways involved in PHA biosynthesis, as explained by [27]. The primary nutritional limitation for PHA accumulation in this study was nitrogen limitation. Nitrogen, which is crucial for microbial growth, affects PHA synthesis, with strains showing varied preferences for nitrogen sources, such as ammonia, urea, yeast extract, and nitrate, as noted by [28], and its deficiency leads to a metabolic shift towards PHA synthesis as a carbon storage material, helping bacteria survive under nutrient stress. This phenomenon is well-documented, with microbes producing PHA under excess carbon and limited nitrogen, sulfur, or phosphorus conditions [28]. For example, *Cupriavidus necator* produced 232 g/L PHB with 82% cellular content in phosphorus-limited conditions [29], and *Rhodospirillum rubrum* achieved 20–46% PHB content under nitrogen-limited, anaerobic, photosynthetic conditions, with acetate or β-hydroxybutyrate as carbon sources [30]. Furthermore, both *Neobacillus niacini* and *Metabacillus niabensis* prefer a neutral pH of 7 for optimal growth and PHA production, aligned with the findings by [19] that indicate that a neutral pH is generally favorable for microbial growth and biopolymer production.

In this study, the optimal shaking speeds for maximizing PHA production were found to be different for the two bacteria: *Neobacillus niacini* performed best at 80 rpm, while *Metabacillus niabensis* was more versatile, achieving peak performance at 150 rpm for both the cell and PHA titer, and at 80 rpm for the best PHA yield relative to the cell dry weight. These observations align with the findings by [31], who noted that the shaking speed impacts oxygen transfer rates, thereby influencing microbial growth and PHA accumulation. With respect to the optimal harvesting time, *Neobacillus niacini* reached its PHA production peak at approximately 24 h, whereas *Metabacillus niabensis* peaked slightly earlier, at approximately 20 h. Yootoum et al. [31] highlighted the importance of this timing, as missing the peak can lead to significantly reduced yields. Bioreactor studies of *Metabacillus niabensis* and *Neobacillus niacini* provide insights into the growth kinetics and metabolic behaviors of these bacteria, under controlled and uncontrolled pH conditions, revealing distinct physiological profiles. For *Metabacillus niabensis*, the uncontrolled pH conditions yielded a PHA volumetric productivity of (0.036 g/L/h) and a specific growth rate of (0.11 h^−1^), slightly lower than the (0.045 g/L/h) reported by [32]. Under the controlled pH conditions, the productivity significantly increased to (1.0 g/L/h), comparable to the (0.9 g/L/h) achieved by *Rhodospirillum rubrum*, as reported by [33]. *Neobacillus niacini*, under uncontrolled pH conditions, showed a PHA volumetric productivity of (0.035 g/L/h) and a specific growth rate of (0.07 h^−1^), similar to the (0.032 g/L/h) for *Cupriavidus necator* [30]. Under the controlled pH conditions, *Neobacillus niacini* productivity increased to (0.13 g/L/h), with a specific growth rate of (0.08 h^−1^), aligned with [29] who reported (0.12 g/L/h). The PHA yield from the substrate (Y_p/S_) for *Metabacillus niabensis* was (0.6 g/g) under the controlled pH, similar to [27] (0.58 g/g), using waste cooking oil. *Neobacillus niacini’s* yield under the uncontrolled pH conditions was (0.25 g/g), comparable to [30], while the biomass yield (Y_x/S_) for *Metabacillus niabensis* was (0.32 g/g) under controlled pH conditions, higher than [28] (0.25 g/g) for *Bacillus megaterium*. These comparisons highlight that both strains exhibit competitive PHA production capabilities, particularly under controlled pH conditions, emphasizing the importance of pH control in optimizing microbial production processes [34,35,36,37,38,39,40].

The FTIR analysis of the PHA samples produced by *Neobacillus niacini* (Sample A) and *Metabacillus niabensis* (Sample B) are consistent with those reported by [28,41], where similar absorption bands were observed in the FTIR spectra of PHAs produced by *Pichia* sp. and *Pseudomonas* spp. The study highlighted a strong absorption band at 1731.26 cm^−1^, assigned to the carbonyl ester bond stretching vibration, along with bands at 3359 cm^−1^ and 3284 cm^−1^ indicating hydroxyl groups. Additionally, our results align with those reported by [37] for PHA produced by *Pseudomonas aeruginosa*, where the FTIR spectra showed main bands at 1726 cm^−1^ for the carbonyl group, and bands in the range of 2925–2852 cm^−1^ for methyl and methylene groups, further confirming the structure of mcl-PHA.

The GC-MS analysis of the PHAs (Figure 11 and Figure 12) produced by *Neobacillus niacini* and *Metabacillus niabensis* provided a detailed breakdown of the monomer units, such as nonanoic acid, hexadecanoic acid, and various dimethyl esters. These results confirm that the produced polymer is a medium-chain-length polyhydroxyalkanoate (mcl-PHA). The common monomers identified include 3-hydroxydecanoate, 3-hydroxydodecanoate, and 3-hydroxyoctanoate. The presence of octanoic and decanoic derivatives further supports the classification as mcl-PHA. Our findings align with the research by [41], who characterized mcl-PHAs produced by Pseudomonas spp. using GC-MS and identified various medium-chain monomers, including 3-hydroxyhexanoate and 3-hydroxyoctanoate, which were also present in our samples.

## 5. Conclusions

This study successfully identified two novel bacterial strains, *Neobacillus niacini* and *Metabacillus niabensis*, which are capable of synthesizing medium-chain-length polyhydroxyalkanoates (mcl-PHAs) from waste cooking oil. Optimal culture conditions were established, with waste sunflower oil and olive oil highlighted as efficient carbon sources. Bioreactor experiments confirmed successful mcl-PHA production, which was verified through FTIR spectroscopy and GC-MS analysis. These findings underscore the potential of utilizing waste materials for sustainable bioplastic production, thus contributing to environmental sustainability and effective waste management solutions. Future research should aim to scale up the production process and explore the mechanical properties of the synthesized mcl-PHAs to facilitate commercial applications.

## Figures and Tables

**Table 1:** PHA titer under optimum experimental conditions.

	*Neobacillus niacini* PHA (g/L)	*Metabacillus niabensis* PHA (g/L)
Carbon Sources		
Sunflower oil	1.1 ± 0.06	0.76 ± 0.01
Waste olive oil	0.13 ± 0.06	0.84 ± 0.05
Nitrogen Sources		
Yeast extract	0.84 ± 0.04	0.33 ± 0.06
Urea	0.38 ± 0.02	0.76 ± 0.01
pH		
pH 7	0.75 ± 0.09	0.73 ± 0.05
Temperature		
35 °C	0.81 ± 0.04	0.77 ± 0.06
Agitation		
100 rpm	1.13 ± 0.12	0.75 ± 0.07
150 rpm	0.5 ± 0.1	0.85 ± 0.07

**Table 2:** Fermentation kinetics of *Neobacillus niacini* during batch fermentation process involving controlled and uncontrolled pH conditions

Fermentation Process	Fermentation Period (h)	Specific Growth Rate (µ)	CDW (g/L)	PHA (g/L)	YP/S (g/g)	Yx/S (g/g)	Biomass Vol. Prod. (g/L/h)	PHA Vol. Prod. (g/L/h)
Uncontrolled pH batch	72	0.07	2	1.75	0.25	0.15	0.02	0.035
Controlled pH batch	72	0.08	4	9.75	0.6	1.62	0.05	0.13

**Table 3:** Fermentation kinetics of *Metabacillus niabensis* during batch fermentation process involving controlled and uncontrolled pH conditions.

Fermentation Process	Fermentation Period (h)	Specific Growth Rate (µ)	CDW (g/L)	PHA (g/L)	Y_P/S_ (g/g)	Y_x/S_ (g/g)	Biomass Vol. Prod. (g/L/h)	PHA Vol. Prod. (g/L/h)
Uncontrolled pH batch	48	0.11	2	1.75	0.17	0.2	0.04	0.036
Controlled pH batch	48	0.11	2.6	4.78	0.6	0.32	0.054	1

## Data Availability

All the authors declare that the data supporting the findings of this study are available within the article.

## Supplementary Materials

The following supporting information can be downloaded at: https://www.mdpi.com/article/10.3390/polym16152150/s1, Figure S1. PHA-producing bacterial strains observed under a fluorescent microscope after screening with Nile Red dye. Figure S2. PHA-producing bacterial strains examined using the Sudan Black staining technique. Figure S3. Extraction of PHA from bacterial strains: (a, b) PHA sheets produced by *Neobacillus niacini* and *Metabacillus niabensis*, respectively, and (c) PHA powder after collection.
