# Supplementary material for: Microbial Biosynthesis of Medium-Chain-Length Polyhydroxyalkanoate (mcl-PHA) from Waste Cooking Oil
Source: Polymers (Basel). 2024 Jul 29;16(15):2150. doi: 10.3390/polym16152150 (PMC11314287; doi:10.3390/polym16152150)
Supplement: Supplementary file 1 [file polymers-16-02150-s001.zip › polymers-3041282-supplementary.pdf]

polymers

MDPI

Supplementary Material

Microbial Biosynthesis of Medium-Chain-Length Polyhydroxyalkanoate (mcl-PHA) from Waste Cooking Oil

Ahmed M. Elazzazy [1,*,†], Khawater Ali Abd [1,†], Noor M. Bataweel [2], Maged M. Mahmoud [2,3] and Afra M. Baghdadi [1]

[1] Department of Biological Sciences, College of Science, University of Jeddah, P.O. Box 80327, Jeddah 21589, Saudi Arabia; 1970136@uj.edu.sa (K.A.A.); amboghdadi@uj.edu.sa (A.M.B.)
[2] King Fahad Medical Research Centre, King Abdulaziz University, Jeddah 21589, Saudi Arabia; no0ora.118@hotmail.com (N.M.B.); mamostafa@kau.edu.sa (M.M.M.)
[3] Department of Medical Laboratory Sciences, Faculty of Applied Medical Sciences, King Abdulaziz University, Jeddah 21589, Saudi Arabia
* Correspondence: amelazzazy@uj.edu.sa
† These authors contributed equally to this work.

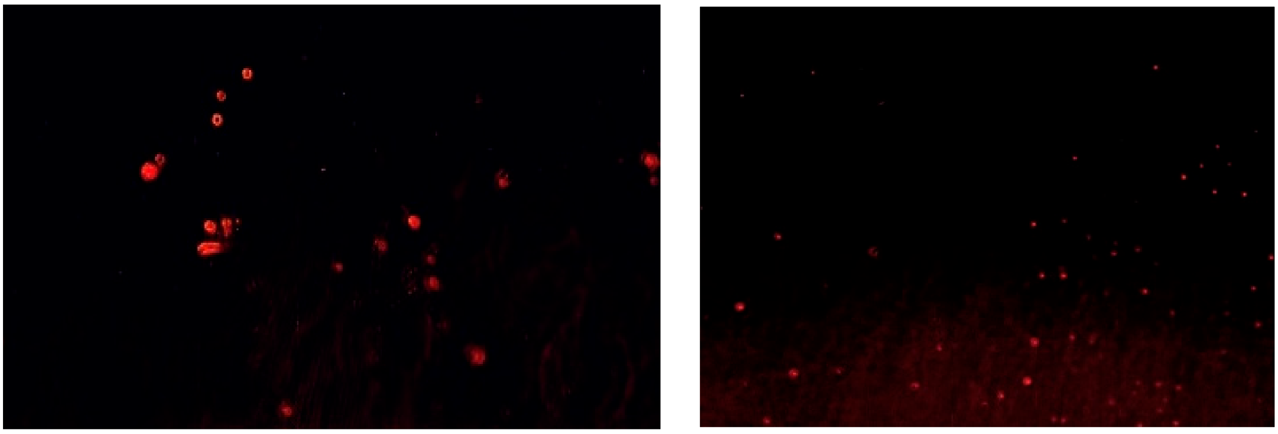

Figure S1. PHA-producing bacterial strains observed under a fluorescent microscope after screening with Nile Red dye.

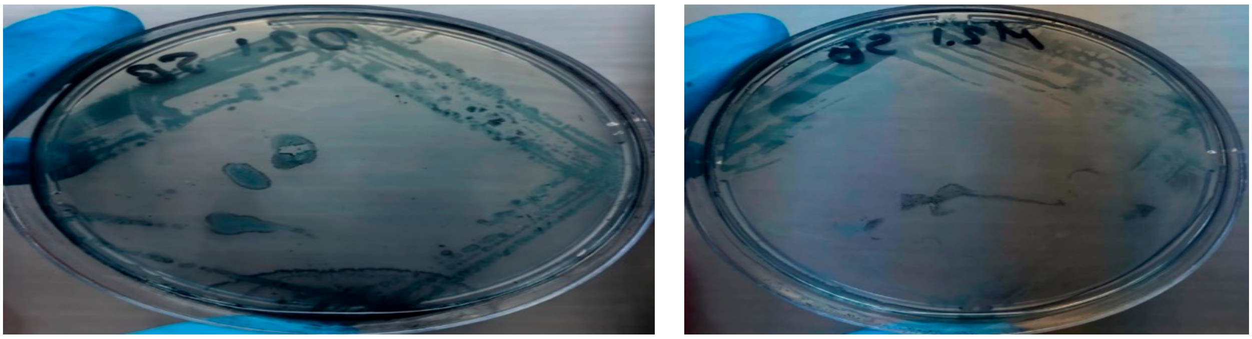

Figure S2. PHA-producing bacterial strains examined using the Sudan Black staining technique.

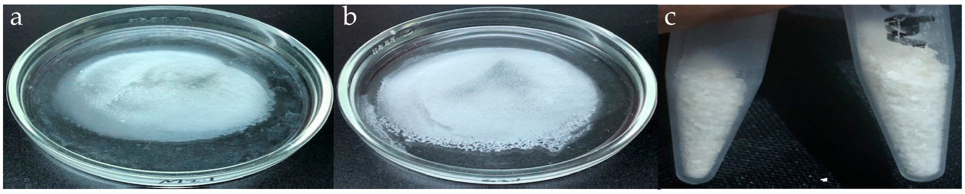

Figure S3. Extraction of PHA from bacterial strains: (a, b) PHA sheets produced by *Neobacillus niacini* and *Metabacillus niabensis*, respectively, and (c) PHA powder after collection.
